# Novel Point Mutations and A8027G Polymorphism in Mitochondrial-DNA-Encoded Cytochrome c Oxidase II Gene in Mexican Patients with Probable Alzheimer Disease

**DOI:** 10.1155/2014/794530

**Published:** 2014-02-18

**Authors:** Verónica Loera-Castañeda, Lucila Sandoval-Ramírez, Fermín Paul Pacheco Moisés, Miguel Ángel Macías-Islas, Moisés Alejandro Alatorre Jiménez, Erika Daniela González-Renovato, Fernando Cortés-Enríquez, Alfredo Célis de la Rosa, Irma E. Velázquez-Brizuela, Genaro Gabriel Ortiz

**Affiliations:** ^1^Laboratorio de Desarrollo-Envejecimiento: Enfermedades Neurodegenerativas, División de Neurociencias, Centro de Investigación Biomédica de Occidente, Instituto Mexicano del Seguro Social, Sierra Mojada No. 800 colonia Independencia 44340 Guadalajara, JAL, Mexico; ^2^Laboratorio de Farmacogenómica y Biomedicina Molecular, Centro Interdisciplinario de Investigación para el Desarrollo Integral Regional, Instituto Politécnico Nacional, Unidad Durango, DGO, Mexico; ^3^Laboratorio de Bioquímica IV, División de Genética, Centro de Investigación Biomédica de Occidente, Instituto Mexicano del Seguro Social, Guadalajara, JAL, Mexico; ^4^Departamento de Química, Centro Universitario de Ciencias Exactas e Ingenierías, Universidad de Guadalajara, Guadalajara, JAL, Mexico; ^5^Departamento de Neurología, Hospital de Especialidades del Centro Médico Nacional de Occidente, Instituto Mexicano del Seguro Social, Guadalajara, JAL, Mexico; ^6^Departamento de Salud Pública, Centro Universitario de Ciencias de la Salud, Universidad de Guadalajara, Guadalajara, JAL, Mexico

## Abstract

Mitochondrial dysfunction has been thought to contribute to Alzheimer disease (AD) pathogenesis through the accumulation of mitochondrial DNA mutations and net production of reactive oxygen species (ROS). Mitochondrial cytochrome c-oxidase plays a key role in the regulation of aerobic production of energy and is composed of 13 subunits. The 3 largest subunits (I, II, and III) forming the catalytic core are encoded by mitochondrial DNA. The aim of this work was to look for mutations in mitochondrial cytochrome c-oxidase gene II (*MTCO II*) in blood samples from probable AD Mexican patients. *MTCO II* gene was sequenced in 33 patients with diagnosis of probable AD. Four patients (12%) harbored the A8027G polymorphism and three of them were early onset (EO) AD cases with familial history of the disease. In addition, other four patients with EOAD had only one of the following point mutations: A8003C, T8082C, C8201T, or G7603A. Neither of the point mutations found in this work has been described previously for AD patients, and the A8027G polymorphism has been described previously; however, it hasn't been related to AD. We will need further investigation to demonstrate the role of the point mutations of mitochondrial DNA in the pathogenesis of AD.

## 1. Introduction

Alzheimer's disease (AD) is a polygenic/complex neurodegenerative disorder in which more than 50 genetic loci are involved [1] and is characterized by a decline in cognitive function, accumulation of extracellular amyloid-*β* peptides (A*β*) and intracellular neurofibrillary tangles, and neuronal loss [2]. Increasing evidence suggests that mitochondrial dysfunction plays an important role in the pathogenesis of AD [3–5]. Mitochondrial DNA (mtDNA) is a circular 16 569 base pair double-stranded DNA, which contains 37 genes, of which 13 genes are crucial subunits of four of the five mitochondrial oxidative phosphorylation (OXPHOS) complexes. The human cytochrome c oxidase is the terminal enzyme complex of the respiratory chain; it is located in the inner mitochondrial membrane where it catalyzes the transfer of electrons from reduced cytochrome c to molecular oxygen. This reaction is coupled with the translocation of protons across the inner membrane, and the electrochemical gradient resulting is used to drive ATP synthesis. Mitochondrial cytochrome c oxidase (MTCO) is made up of 13 subunits; its catalytic core is made up from the protein products of the *MTCO I*, *II*, and *III* genes that are encoded in mitochondrial DNA [6]. Therefore, these genes play key roles in the regulation of cellular energy production. *MTCO II* gene is located between nucleotides 7586 and 8294 of mitochondrial DNA and codified for a protein of 227 amino acids [7].

Diminished cytochrome c oxidase activity has been described in AD postmortem cerebral cortex [8–11] and platelets [12]. In addition, data obtained by Parker et al. [10] and Cardoso et al. [12] suggested that cytochrome c oxidase protein levels are normal in AD brain and platelets, respectively. However, Kish et al. [8] using an immunohistochemistry approach reported a reduced level of this protein complex in AD brain. These data could suggest that brain cytochrome oxidase is kinetically abnormal; thus, this dysfunction must arise from a catalytic defect rather than an underproduction [9]. However, mutations of the mitochondrial genome are widely recognized as important causes of disease [13]; therefore, the aim of the present work was to look for mutations in cytochrome c oxidase gene II in blood samples from probable AD Mexican patients.

## 2. Material and Methods

### 2.1. Patients

AD subjects were recruited from the Clínica de Trastornos Cognitivos y Demencias at the Hospital de Especialidades del Centro Médico Nacional de Occidente at Guadalajara, Jalisco, México. This study was approved by the Comisión Nacional de Investigación Científica Review Board and was carried out in accordance with the principles of the Declaration of Helsinki as revised in 2000. Informed consent was obtained from the patient or caregiver (when indicated) by a staff physician. Patients fulfilled the NINCDS-ADRDA criteria for probable AD. They did not manifest signs or symptoms of any alternative neurodegenerative disorder. Twenty patients were male and thirteen were female; the oldest subject was 86 years old and the youngest was 42 years old. Seven patients were siblings of three not-related families. Nineteen probable AD subjects were under 60 years of age (early-onset group); within that group, fourteen subjects had familial history of AD. Other fourteen probable AD subjects were above 60 and were part of the late-onset group; they had no familial history of AD.

### 2.2. DNA Extraction

Blood samples from 33 patients with probable AD were collected in tubes containing potassium EDTA by a trained physician. DNA was obtained from 10 mL of blood following the Miller technique [14] and purified by ethanol precipitation and its concentration was determined by spectrophotometry UV absorption at 260 nm/280 nm.

### 2.3. PCR and Sequencing

The *MTCO II* gene was amplified by PCR using the following primers 5′-CAAGCCAACCCCATGGCCTCC-3′ and 5′-AGTATTTAGTTGGGGCATTTCAC-3′ as reported elsewhere [15] by using the *Big Dye Terminator v 3.0 cycle sequencing ready reaction* sequencing kit (Perkin-Elmer) according to the manufacturer's instructions. DNA fragments were analyzed on a DNA sequencer and ABI Prism 310 Genetic Analyzer (Applied Biosystems). Each DNA fragment was sequenced in both sense and antisense directions.

## 3. Results

**Figure 1:**
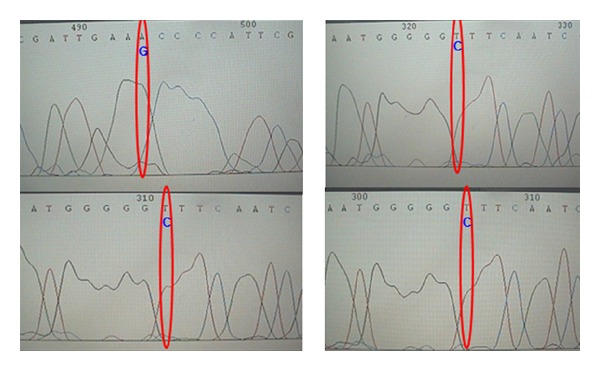
*Sequencing histograms of the mitochondrial MTCOII  gene*. Representation of the sequencing in *MTCO  II* gene. Variations are circled in red and nucleotide change is shown in blue.

Analysis of mitochondrial cytochrome c oxidase II gene obtained from blood samples revealed that 4 nonrelated patients with probable AD harbored the polymorphism A8027G (three of them were diagnosed with late onset Alzheimer's disease, with familial history of the disease and one of them with early onset Alzheimer's disease) (Tables 1 and 2). This polymorphism shows a frequency of 12% in the analyzed sample. The amplification and sequencing of the mitochondrial cytochrome c oxidase II gene of these four patients are shown in Figure 1.

**Figure 2:**
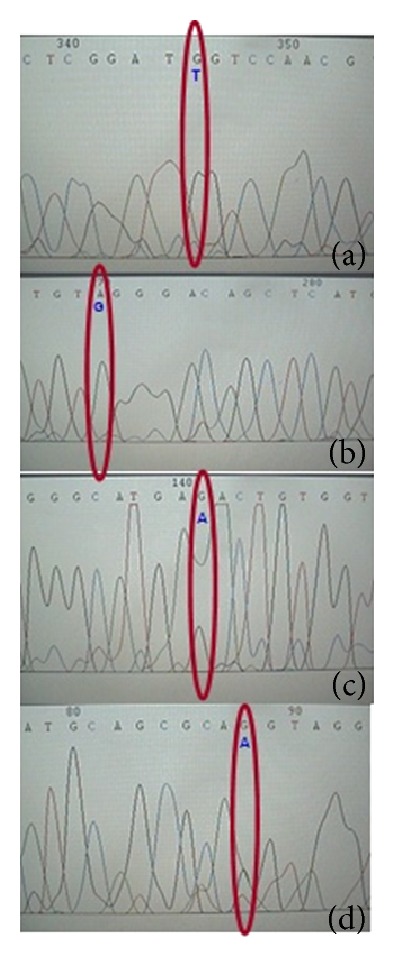
*Schematic representation of the four Mexican patients*. In this figure is presented point mutation by sequencing. (a) represents the mutation A8003C, (b) represents the mutation for T8082C, (c) represents C8201T, and (d) represents G7603A.

Four patients with EOAD harbored only one of the following point mutations: A8003C, T8082C, C8201T, and G7933A, respectively (Table 3). The results of the sequencing of the point mutations found in the 4 EOAD patients are shown in Figure 2. The A8003C transversion converts a polar neutral asparagine residue to a basic histidine. The T8082C transition converts proline to a leucine. C8201T transition converts a nonpolar aromatic phenylalanine to a nonpolar leucine. G7603A transversion converts a basic arginine to a basic histidine.

From the 33 patients that were enrolled for this study only 8 of them presented a nucleotide variation in *MTCO II* gene, representing the 24% of the total of the patients.

Three of the eight patients (37.5%) showed nucleotide variation and were diagnosed with LOAD. While, five of the eight patients (62.5%) were EOAD.

## 4. Discussion

Point mutations in *MTCO II* gene have been reported in several neurodegenerative diseases [16–18] and cancer [19]. However, the significance of gene mutations in AD pathogenesis still remains controversial [20, 21]. Previously, Davis et al. [15] identified three heteroplasmic mtDNA point mutations in *MTCO II* gene in patients with sporadic Alzheimer's disease. However, this finding has been attributed to an artifactual PCR amplification of “mtDNA-like” sequences embedded in nDNA (“mtDNA pseudogenes”), since the boiling method used preferentially releases nDNA relative to mtDNA, thereby enhancing the ability of PCR to amplify such pseudogenes, resulting in an erroneous interpretation of mtDNA heteroplasmy [13, 22]. On the other hand, it has been reported in a patient diagnosed as definitive Alzheimer that the following missense mutations are present: G8206T and A8224T [23]. In this study, we were unable to find the mutations reported previously by Davis et al. [15] and Qiu et al. [23]. However, we found four novel mutations in *MTCO II* gene. To our knowledge, none of these mutations were found in other neurodegenerative diseases.

MTCO II protein is the third largest subunit of the cytochrome c oxidase complex and plays an essential role in respiration. It is the sole binding partner with cytochrome c and is the first recipient of its electrons. These electrons initially pass to the Cu, site of cytochrome c oxidase, which is composed of two copper atoms coordinated with six ligands (two cysteines, two histidines, a methionine, and a peptide carbonyl of a glutamate), all contributed by MTCO II. The glutamate ligand at the CuA site plays a dual role in that its side chain also is a ligand (along with two residues from MTCO I) for a magnesium atom. This Mg center probably performs a role in the transfer of electrons to MTCO I [6]. It could argue that the mutations found in this work modified the protein conformation, due to the different physicochemical characteristics of replaced amino acids. That conformational change could alter the electrons binding site or the electrons transfer and this contributes to a diminution in enzymatic activity. The diminished cytochrome c activity could cause a decrease in ATP synthesis and diversion of electrons from their normal pathway resulting in increasing in superoxide radical production. Thus, MTCO alterations can lead to increased reactive oxygen species generation, oxidative damage to mitochondrial membranes, and increased vulnerability to excitotoxins and may be important in the pathogenesis of AD [24].

The polymorphism A8027G found in 4 of the Mexican patients enrolled in this study has not been reported elsewhere in any neurodegenerative disease. This polymorphism, converts a hydrophobic alanine to a hydrophilic threonine, caused by a transition located in the first position of the codon and associated with reversal of alanine (Ala) to threonine (Thr). At this regard, Ala contains a normal Carbon *β*, meaning that it is generally hindered with respect to the conformations that the backbone can adopt. Furthermore, the Ala side chain is very non-reactive and is rarely directly involved in protein function. However, it can play a role in substrate recognition or specificity. In contrast, Thr is a slightly polar amino acid and contains a Carbon *β* branched. This means that there is a lot more bulkiness near to the protein backbone, and thus Thr is more restricted (than Ala) in the conformations the main-chain can adopt.

The point mutations that we report have not been related to any other disease; the previous nucleotide variations have been searched on GeneBank and MitoMap, where only the A8027G has been described as an SNP, while the other 4 point mutations found in 4 Mexican patients have not been described previously [25, 26]. This study requires future research for patients with AD of early onset which could present the reported mutations.

## Figures and Tables

**Table 1:** Polymorphism A8027 in Alzheimer's disease patients.

Data obtained	Nucleotide wild type	Single nucleotide polymorphism	Effect	Number of patients
A8027G	G	A	Nonsynonymous substitution	4

**Table 2:** Patient's characteristics with the A8027G polymorphism.

Patient	Gender	Age	Patient diagnosis	Familial history
Subject 1	Female	76	Late onset	yes
Subject 2	Male	50	Early onset	no
Subject 3	Male	73	Late onset	yes
Subject 4	Female	66	Late onset	no

**Table 3:** Novel point mutations and patient's characteristics.

Number of patients	Mutation	Sex	Age	Patient diagnosis
1	G7603A	Male	49	Early onset
1	A8003C	Female	44	Early onset
1	T8082C	Male	44	Early onset
1	C8201T	Male	57	Early onset
